# Cutaneous Eruptions

**Published:** 1875-08

**Authors:** 


### CUTANEOUS ERUPTIONS.

We spoke favorably of the effect of tar in affections of the skin last month, and referred especially to the convenient means of applying it in concentrated form, through the use of "Packer's Tar Soap." Our article has been productive of one result, at least; it has brought the subject up for broad discussion, and has flooded us with letters accordingly. We cannot reply to five or six hundred correspondents in detail. Our space is too limited to be filled with conundrums and their solutions, however entertaining these might be to a few. Every line counts with us, and we owe it to ourselves to see that not a line is wasted. We are not bothered to find matter for our pages. What troubles us is to get in a tenth part of the live matter which accumulates from month to month. Next year, if all goes well with us, we will enlarge the JOURNAL and considerably improve it. With sixteen pages added, we shall hope to devote a little more space to the letters of our friends. The added room will permit us to insert a devitalized item now and then, or to copy more largely from other publications. At present, we are compelled to boil everything down, to desiccate the mental food which we serve up. Our readers will notice that we often answer their private letters in our pages, without even stating that a communication has been received, or even repeating the query. This will explain some things to our correspondents, and especially to the multitude who have written us on this tar topic and have received no response. In another place we answer some of the suggestions alluded to.

